# Endoscopic nasobiliary drainage-based saline-injection ultrasound: an imaging technique for remnant stone detection after retrograde cholangiopancreatography

**DOI:** 10.1186/s12876-022-02394-8

**Published:** 2022-06-27

**Authors:** XiaoDong Wu, ShuoDong Wu, ShaoShan Tang

**Affiliations:** 1 Department of General Surgery, Shengjing Hospital of China Medical University, Shenyang, 110004 China; 2 Department of Ultrasonography, Shengjing Hospital of China Medical University, Shenyang, China

**Keywords:** ERCP remnant stone, ENBD, Ultrasound

## Abstract

**Background:**

The purpose of this retrospective study aimed to assess the accuracy of detection of remnant common bile duct (CBD) stones by injecting saline through endoscopic nasobiliary drainage (ENBD) tubes under transabdominal ultrasound (US) guidance.

**Method:**

Stone extraction and ENBD are regularly achieved through endoscopic retrograde cholangiopancreatography (ERCP) in patients with CBD stones. At 1–3  days thereafter, routine US studies were performed and repeated, using ENBD tubal saline injections (20–100  mL).

**Results:**

A total of 302 patients underwent standard ERCP stone extractions in conjunction with occlusion cholangiograms, routine US testing, and ENBD-based saline-injection US exams. By occlusion cholangiogram, remnant stones were suspected in 31 (10.3%) patients in total of 302, and 26 (83.8%) were verified as true positives (sensitivity, 50.9%; specificity, 98.0%). Routine US studies proved suspicious in 13 (4.3%) patients in total of 302, and 12 (92.3%) were verified as true positives (sensitivity, 23.5%; specificity, 99.6%). Using ENBD-based saline-injection US, suspected stones were identified in 50 (16.6%) patients in total of 302, and 46 (92%) were verified as true positives (sensitivity, 90.1%; specificity, 98.4%). The sensitivity of ENBD-based saline-injection US significantly surpassed that of occlusion cholangiogram (*p* < 0.001) and routine US (*p* < 0.001).

**Conclusion:**

Detection of remnant CBD stones via ENBD-based saline-injection US is a valid, inexpensive, and repeatable means of patient screening that is non-invasive, radiation-free, and dynamically informative. This may help improve the accuracy of detecting remnant CBD stones after ERCP.

**Supplementary Information:**

The online version contains supplementary material available at 10.1186/s12876-022-02394-8.

## Background

Choledocholithiasis is a common condition that undermines human health, affecting 10–20% of patients with biliary tract disease [1, 2]. Endoscopic retrograde cholangiopancreatography (ERCP) is widely accepted for the diagnosis and treatment of choledocholithiasis. In addition to eliminating the need for surgery, the minimal trauma entailed, short procedural times, few complications, and brief hospital stays are clearly advantageous [3, 4]. Standard methods of ERCP primarily include sphincterotomy (EST), balloon dilation, balloon lithotripsy, basket retrieval, endoscopic nasobiliary drainage (ENBD), and occlusion cholangiogram. Occlusion cholangiograms are often used to determine the completeness of stone clearance. However, a previous study has shown that they fail to detect residual calculi in 11–30% of cases, especially in the setting of a severe pneumobilia or biliary ductal dilatation and in the course of lithotripsy [5–8]. Currently, rates of recurrent choledocholithiasis after ERCP range from 4–24%. Residual stones are important causative factors in such recurrences [9–11], prompting recrudescence of biliary symptoms, complications (i.e., pancreatitis, cholangitis), and clinical issues, including untoward hospital costs and patient suffering [2, 5].

Aside from occlusion cholangiogram (mentioned above), current imaging methods for post-ERCP detection of residual stones also include computed tomography (CT), ultrasound (US), magnetic resonance cholangiopancreatography (MRCP), and trans-ENBD cholecystography. [12] These techniques are mainstays of clinical practice. However, each has its drawbacks, contributing to incorrect or missed diagnoses, and none of them separately excels for this purpose. A new imaging approach, combined with traditional methodology, might therefore be beneficial in terms of detecting residual stones.

For this study, we used ENBD access to our advantage in formulating a diagnostic application. ENBD is a widely used therapeutic tool in ERCP procedures to relieve obstruction. Once biliary outflow is re-established, ductal pressure, jaundice, and inflammation are reduced. Our intent was to cultivate imaging advancement through the innovative use of ENBD and transabdominal US by injecting ENBD lines with saline under US guidance, thus enhancing visualization of the biliary tract and aiding in post-ERCP detection of remnant stones. Thus, we aimed to assess the accuracy of detecting remnant CBD stones by injecting saline through ENBD tubes under transabdominal US guidance. Previous study have preliminarily introduced the application of ENBD saline injection US in the diagnosis of post ERCP residual CBD stones [13]. However, due to the limitation of the number of cases and lack of the data, there are still exist many question about ENBD saline injection US need futher conformation.

## Materials and method

### Patient selection

This retrospective study was approved by the local ethics committee of the Affiliated Shengjing Hospital of China Medical University and written informed consent from each patient was waived by the local ethics committee of the Affiliated Shengjing Hospital of China Medical University in the ethical approval and consent to participate section in manuscipt.. Between January 2015 and June 2019, all patients diagnosed with CBD stones (based on clinical manifestations and imaging) treated by endoscopic extraction/ENBD at the Department of Second General Surgery, Shengjing Hospital of China Medical University, were eligible for study. EST was warranted in each patient, and none had severe cardiopulmonary dysfunction, iodinated contrast allergy, or coagulation defects. Operative risks, postoperative complications, and the potential for remnant stones were explained, and signed consent was obtained in advance of procedures. Participating patients were adults > 18 years old, capable of granting written informed consent. Intrahepatic duct stones, malignancies, choledochol cysts, primary sclerosing cholangitis, or anatomic alterations (i.e., Billroth II, Roux-en-Y gastric bypass, or Whipple procedures) were grounds for exclusion. Baseline characteristics and procedural parameters of study enrollees are shown in Table 1.

**Table 1:** Baseline characters of included patients

		Occlusion cholangiogram/Routine Ultrasound/ENBD Injection ultrasound (*n* = 302)
	Age (year)	58.1 ± 15.6
Gender	Male	154(51%)
	Female	148(49%)
BMI (kg/m^2^)		27.7 ± 0.7
History of cholecystectomy		77(27%)
Gallbladder stone		193 (64%)
Hepatolith		39 (13%)
Treatment method during ERCP	EST	287(95%)
	Balloon Dilation	214(71%)
	Balloon extraction	220(73%)
	Basket retrieval	88 (29%)
	Lithotripsy	18 (6%)
ERCP duration (min)		26 ± 8.6
LABS	Total bilirubin (umol/L)	57.5 ± 6.0
	Direct bilirubin (umol/L)	37.4 ± 4.8
	Indirect bilirubin (umol/L)	20.1 ± 2.0
	Alkaline phosphatase (U/L)	166.5 ± 10.8
	ALT (U/L)	151.0 ± 17.1
	AST (U/L)	103.5 ± 14.0

### ERCP procedure and post ERCP detection

ERCP was performed by endoscopic specialists with at least associate professor status and a roster of > 500 surgical cases. The standard ERCP protocol was applied, including biliary cannulation and cholangiography to delineate existing biliary stones. (OLYMPUS tjf260v, BeiJing, China)Once identified, the stones were removed by conventional means, namely EST, balloon extraction, basket retrieval, or lithotripsy. Iodinated contrast studies were then conducted to inspect the remnants. In the presence of substantial filling defects, extractions were repeated until no remnant stones were evident on occlusion cholangiogram. Flush the bile duct with saline repeatedly to clean up the debris caused by lithotripsy. Concentrations of contrast were at the discretion of the individual endoscopists. ENBD cannulas were then placed, and routine transabdominal US studies were performed. US examinations involving ENBD tubal injections of saline and trans-ENBD cholecystography were performed 1–3 days after ERCP. Routine US studies were conducted first by physicians specializing in ultrasonography with professor status to check all CBDs and look for remnant stones. Afterwards, 20–100 mL of saline was slowly injected via the ENBD tube to avoid formation of bubbles. The studies were subsequently repeated to screen for remnant stones, comparing bile duct images before and after saline injection. Patient vital signs and any discomfort were duly monitored, as were ultrasound findings (i.e., ranges of CBD diameters/lengths and detection rates of CBD remnant stones). US examination was performed using the same US machine (i.e., LOGIQ E9 US scanner; GE Medical Systems, Milwaukee, WI, USA). Residual stones larger than 5 mm are considered to be meaningful to aviod the statistical error induced by debris generated after lithotripsy operation. Any residual calculi suspected by transabdominal US or trans-ENBD cholecystography were cause for second-round extraction. Stones removed during secondary ERCP treatments or surgical interventions were considered remnants.

### Statistical analysis

SPSS software (version 20.0 for Windows; SPSS Inc., Chicago, IL, USA) were used to perform statistical analyses. Quantitative data (i.e., patient age and CBD length) were expressed as mean ± standard deviation, which were compared by Student's *t*-test. *χ*2 test or Fisher's exact probability test was used to analyze categorical variables. And *p* < 0.05 was considered a significant difference.

## Results

In this study, remnant CBD stones were defined as those removed during secondary ERCP or surgical interventions. Of the 311 patients in whom standard ERCP stone extraction occurred, 9 were excluded due to complications of malignant tumors or other diseases. In addition to standard ERCP lithotomies, the remaining 302 were subjected to occlusion cholangiograms, routine postoperative US imaging, and ENBD-based saline-injection US studies.

**Fig. 1:**
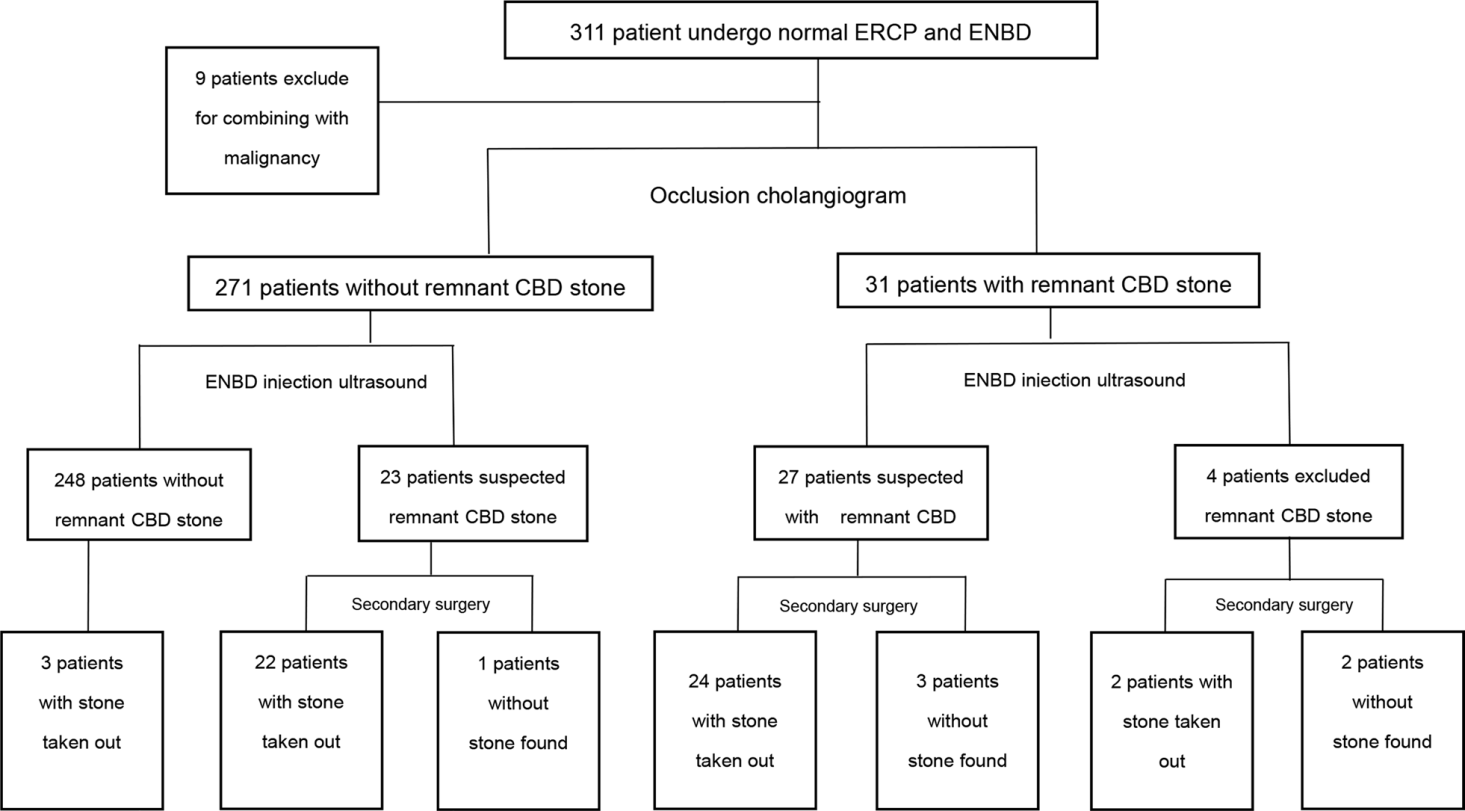
The examination results of patients

**Fig. 2:**
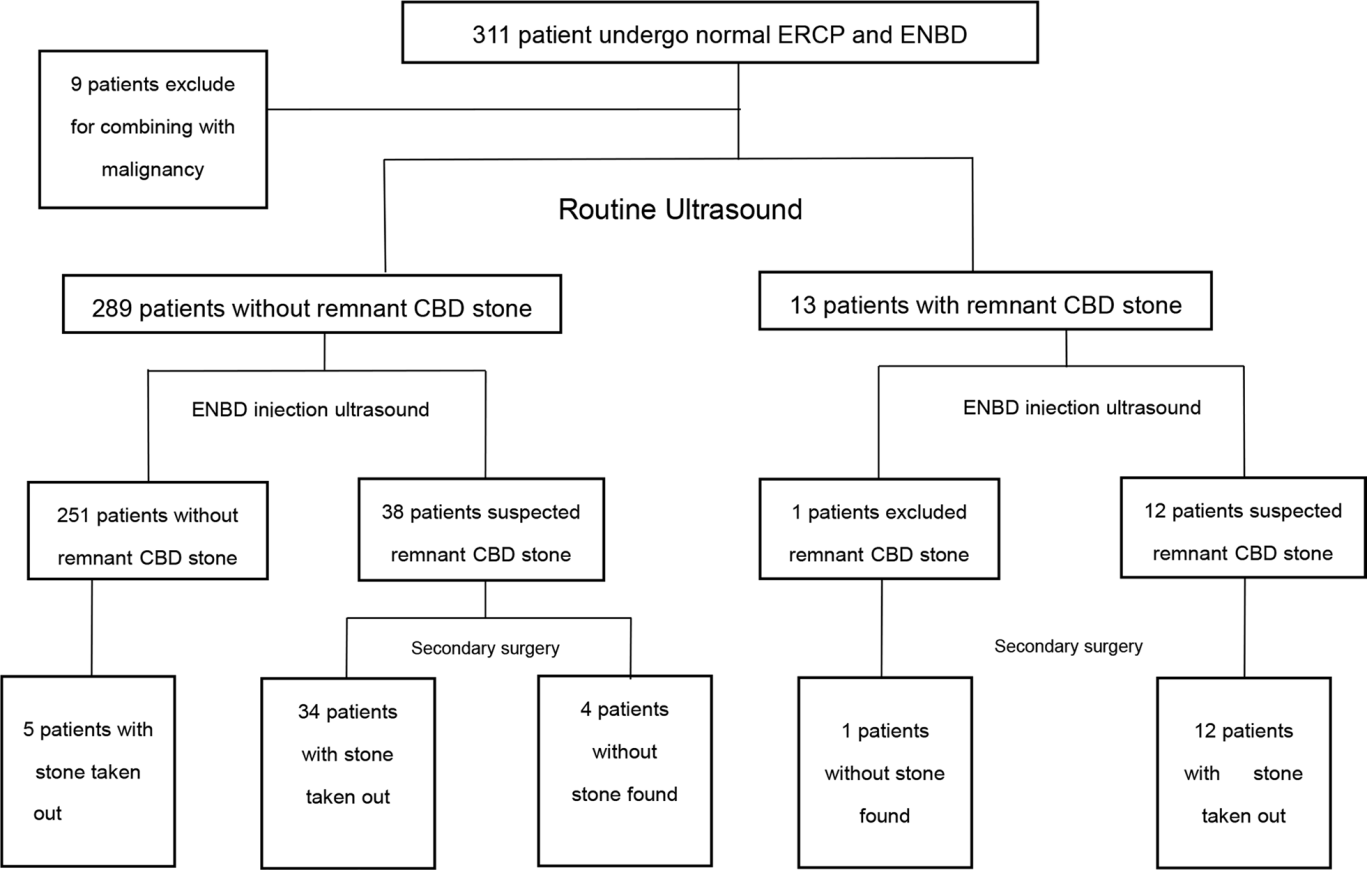
The examination results of patients

During routine ERCP, suspected residual stones were seen by occlusion cholangiogram in 31 of the 302 qualifying patients, the vast majority (*n* = 271) showing none. Conventional US studies were also suspicious of residual stones in 13 of these 31 patients (none identified in 18). Within the group of 31 patients (cholangiogram-positive), residual stones were evident in 28 patients using ENBD-based saline-injection US (no stones in 3). Ultimately, 26 of these 31 patients underwent stone extractions during the secondary ERCP procedures. (Fig. 1) In the 13 patients with suspected remnants by routine US, 12 had stones removed via secondary ERCP. In all 271 patients with negative cholangiograms, routine US images were negative for residual stones as well. However, ENBD-based saline-injection US studies were suspicious of residual calculi in 22 of these patients. When subjected to secondary treatment, stones were eventually extracted from 20 of these 22 patients. It is worth mentioning that among the patients (*n* = 249) who proved negative for residual stones in all three modes of testing (occlusion cholangiography, routine, and saline-injection US), three had stones removed by secondary lithography. This is because even though the imaging results were normal, they had clinical manifestations such as abdominal pain, jaundice, and elevated bilirubin (Fig. 2).

**Fig. 3:**
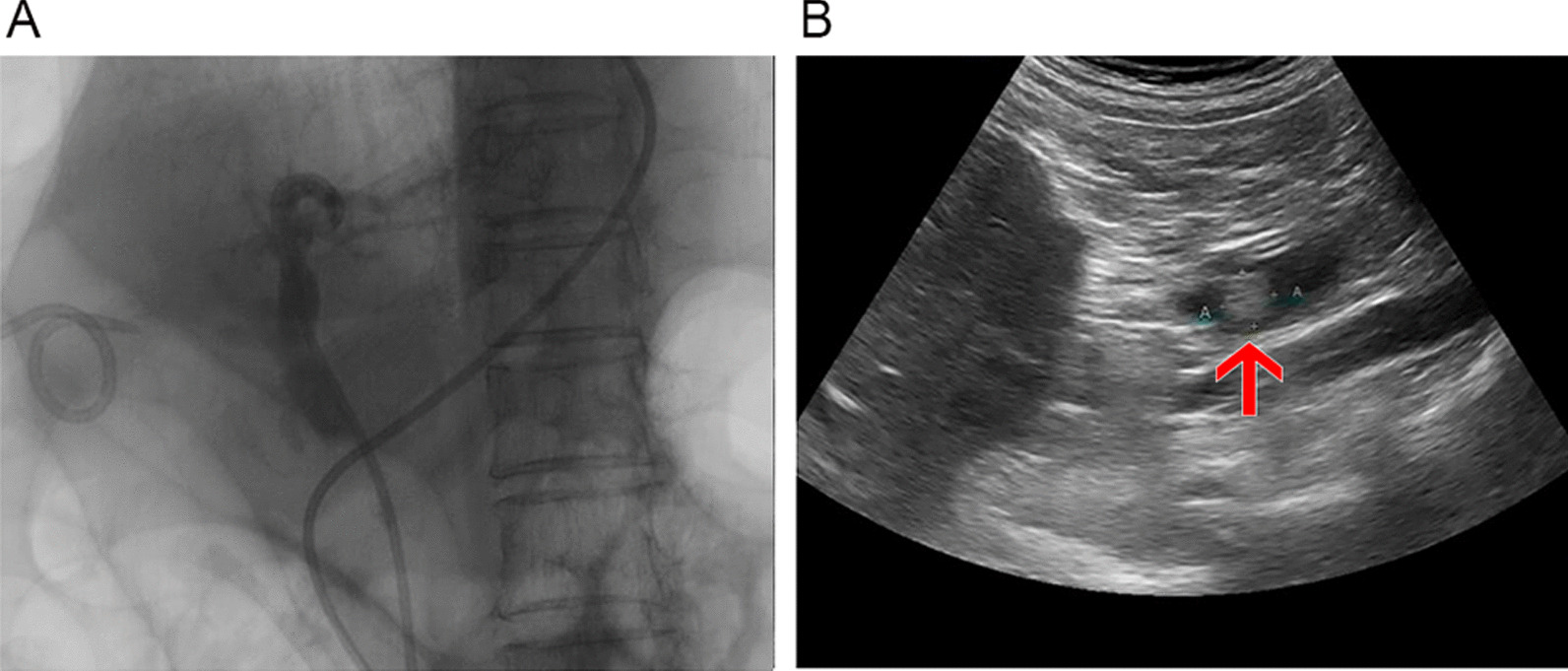
Comparison between occlusion cholangiogram and ENBD saline injection in ultrasounds in a 78-year-old women**. A**. There are no significant stones found in occlusion cholangiogram. **B**. Remnant stone is found by ENBD saline injection ultrasound

Of the 302 patients studied, there were 26 true positives, 246 true negatives, 5 false positives, and 25 false negatives by occlusion cholangiography (specificity, 98.0%; sensitivity, 50.9%; false-positive rate, 2.0%; false-negative rate, 49.1%). By comparison, ENBD-based saline-injection US imaging yielded 46 true positives, 247 true negatives, 4 false positives, and 5 false negatives (specificity, 98.4%; sensitivity, 90.1%; false-positive rate, 1.6%; false-negative rate, 9.9%), indicating significantly greater sensitivity (*p* < 0.001) and a significantly lower false-negative rate (*p* < 0.001). The two examinations did not differ significantly (*p* = 0.689) in terms of specificity and false-positive rate; although imaging of CBD length by saline-injection US performed better than that of occlusion cholangiography (*p* < 0.001), CBD diameter showed no statistical difference (*p* = 0.601) (Table 2). A comparison of imaging effects between occlusion cholangiography and ENBD-based saline US is shown in Fig. 3.

**Table 2:** Comparison of the results of occlusion cholangiogram and ENBD saline injection ultrasound

		Occlusion cholangiogram	ENBD Injection ultrasound	*p*-value
Common bile duct	Length (mm)	53.4 ± 1.50	62.62 ± 1.17	< 0.001
	Diameter (mm)	10.63 ± 0.474	10.36 ± 0.369	0.601
True positive individual		26	46	< 0.001
True negative individual		246	247	0.689
False positive individual		5	4	0.689
False negative individual		25	5	< 0.001
Specificity		98.0%	98.4%	0.689
Sensitivity		50.9%	90.1%	< 0.001
False positive rate		2.0%	1.6%	0.689
False negative rate		49.1%	9.9%	< 0.001
Estimated accuracy		90.1%	96.4%	< 0.001

**Fig. 4:**
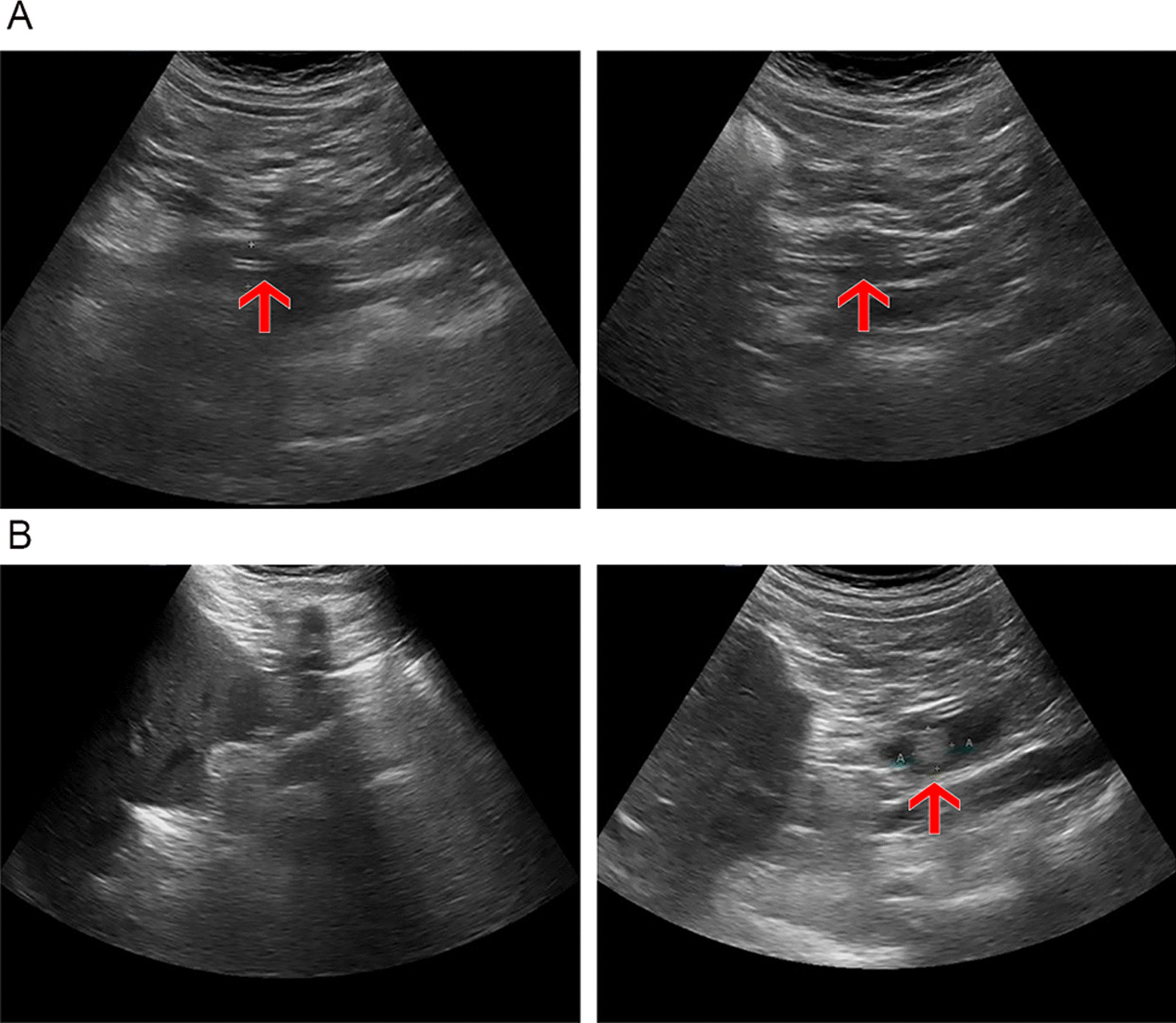
Comparison between routine ultrasound and ENBD saline injection ultrasound in a 78-year-old women. **A**. The imaging length of CBD in ENBD saline injection ultrasound (right) is significantly longer than in routine ultrasound (left). **B**. Remnant stone in ENBD saline injection ultrasound found (right) that cannot be found in routine ultrasound (left)

In routine US examinations of the 302 patients studied, there were 12 true positives, 250 true negatives, 1 false positive, and 39 false negatives (specificity, 99.6%; sensitivity, 23.5%; false-positive rate, 0.4%; false-negative rate, 76.5%). Again, ENBD-based saline-injection US significantly outperformed routine US, demonstrating higher sensitivity (*p* < 0.001) and a lower false-negative rate (*p* < 0.001), whereas specificities and false-positive rates did not differ significantly (*p* = 0.249). CBD imaging rates (*p* < 0.001), lengths (*p* = 0.048), and diameters (*p* = 0.034) also differed significantly (Table 3), with higher magnitudes displayed by saline-injection US. A comparison of the imaging effects of routine US and ENBD-based saline US is shown in Fig. 4.

**Table 3:** Comparison of water injection results between normal ultrasound and ENBD saline-injection ultrasound

		Routine ultrasound	ENBD Injection ultrasound	*P* value
Common bile duct	Length (mm)	38.05 ± 1.87	62.62 ± 1.17	0.048
	Diameter (mm)	6.959 ± 0.258	10.366 ± 0.369	0.034
Imaging degree of common bile duct	without imaging of Whole bile duct	87(28.8%)	7(2.3%)	< 0.001
	without imaging of upper part	71(23.5%)	7(2.3%)	< 0.001
	without imaging of lower part	136(45.0%)	15(4.9%)	< 0.001
True positive individual		12	46	< 0.001
True negative individual		250	247	0.249
False positive individual		1	4	0.249
False negative individual		39	5	< 0.001
Specificity		99.6%	98.4%	0.249
Sensitivity		23.5%	90.1%	< 0.001
False positiverate		0.4%	1.6%	0.249
False negative rate		76.5%	9.9%	< 0.001
Estimated accuracy		86.7%	96.4%	< 0.001

## Discussion

In dealing with CBD stones, especially those involving lower CBD segments, ERCP treatment has increasingly been accepted by the majority of doctors and patients [14–16]. There is substantial evidence that endoscopic stone extraction may reduce the risk of postoperative complications associated with traditional open surgery, lessening surgical trauma, shortening hospitalization times, and relieving pain through long-term biliary drainage tube placement [4–6]. However, if some remnants are retained during less than full endoscopic CBD visualization, acute suppurative cholangitis and acute pancreatitis may rapidly ensue. Consequently, the search for an effective diagnostic method that helps avoid such scenarios has important clinical ramifications [17, 18]. For this study, we used ENBD access to our advantage in formulating a diagnostic application. ENBD is a treatment method applicable to acute suppurative obstructive cholangitis, bile duct obstruction caused by primary or secondary tumors, bile duct obstruction caused by hepatolithiasis, prevent incarceration of common bile duct stones, biliary pancreatitis, benign stricture of bile duct, traumatic or iatrogenic bile fistula, and sclerosing cholangitis [19]. ENBD also been a widely used therapeutic tool in ERCP procedures to relieve obstruction.

MRCP and EUS are also widely applied in CBD stone diagnosis, and various kinds of clinical guidelines have confirmed their superior positions [20]. However, they are currently not fit for ERCP residual CBD stone detection. For ERCP residual stone detection, the examination method should not only meet the requirement of exact CBD stone detection, but should also meet the requirements of economical, simple procedures causing less suffering of patients during multiple examinations in a short period of time. Thus, the high cost of MRCP and increase of suffering by EUS constitute limitations of these methods in application.

An occlusion cholangiogram is the acknowledged gold standard for the diagnosis of bile duct stones. However, various studies have documented that this method may at times lead to misdiagnosis. In instances of pneumobilia or lithotripsy during ERCP, the chances of misdiagnosis are significantly increased. Intestinal inflation/dilation in the course of ERCP and pneumobilia due to retrograde biliary migration of intestinal gas after EST may impact the diagnostic performance. In addition, small stones that persist after lithotripsy are often missed by cholangiography. In this study, reference to previous literature, residual stones larger than 5 mm are considered to be meaningful, [21] because the debris generated after lithotripsy operation may increase detection rate and cause statistical error in each examination method (Additional file 1: TableS1). According to the result, the sensitivity of occlusion cholangiogram (via ENBD) for detecting post-ERCP remnant stones was only 50.9%, and the false-negative rate was 49.1%.It is worth noting that in this study, among the 271 patients without stones found by intraoperative occlusion cholangiogram, 23 patients were suspected to have residual stones after ENBD saline injection ultrasound examination, and 22 patients were removed stones during the secondary ERCP or surgical interventions. It was proved that the residual stones of these 22 patients were missed by intraoperative occlusion cholangiogram and found by ENBD saline injection ultrasound. In addition, intraoperative occlusion cholangiogram has its own limitations. For example, CT imaging of pigment stones is not ideal, owing to inherent image-forming principles, and gas interference may lead diagnosis astray. Furthermore, fever, biliary tract infection, and radiation injury related to procedural injection of contrast are typically disadvantageous, whereas saline-injection US effectively averts such problems. Moreover, in pregnant women and other special-needs patients who are ill-suited for occlusion cholangiogram, saline-injection US constitutes a viable alternative option.

Routine US studies are not very useful for diagnosing choledocholithiasis or assessing a normal CBD. Particularly in the lower CBD segment, interference by the intestinal tract and field gas creates a suboptimal environment. In addition, the sphincter of Oddi is functionally altered after EST, allowing retrograde biliary migration of intestinal gas. The limited space between the ENBD cannulas and the CBD wall hinders ductal visualization, so any small stones harbored within cannot be detected. In this study, the sensitivity of routine US imaging for identifying residual stones was 23.5%, the rate of complete CBD imaging was only 28.8%, and the imaging rate of lower-segment CBD was 45%. Thus, we used a novel approach of injecting saline via ENBD tubes (under US guidance) to disengage the ENBD/CBD interface, creating more space and expelling any biliary tract gas, thereby overcoming the related drawbacks.In this study, among the 289 patients without stones found by routine ultrasound, 38 patients were suspected to have residual stones after ENBD saline injection ultrasound examination, and 34 patients were removed stones during the secondary ERCP or surgical interventions,which indicating that the residual stones of these 34 patients were missed by routine ultrasound and found by ENBD injection ultrasound. US examinations have the advantage of being real-time and dynamic in nature. In our study, patients were regularly placed in left lateral positions, making it easier for retrograde CBD gas to be discharged into the intestinal tract and preventing its return. At the same time, injection of saline served to eliminate gas in the duodenum and enhance delineation of the lower CBD. The results of this study show that, the length and diameter of the common bile duct of ENBD saline injection ultrasound are higher than that of routine ultrasound, which proves that water injection ultrasound can make up for the shortcoming of poor imaging effect of bile duct during routine ultrasound.

ENBD-based saline-injection US is simple, convenient, and inexpensive, and may also significantly improve the quality of US examinations and accuracy of CBD stone detection, thus aiding in therapeutic guidance during follow-up. Under this novel approach, allergy to iodinated contrast and radiation exposure during ENBD biliary angiography is no longer an issue, and patients find its cost (which undercuts that of trans-ENBD cholangiography) more acceptable. The sensitivity, specificity and estimated accuracy of ENBD-base saline injection US for the detection of residual CBD stones were 90.1%, 98.4% and 96.4%, respectively. These figures are significantly better than those achieved by occlusion cholangiography or conventional US, as were CBD length and diameter imaging outcomes. Thus, this strategy seems to significantly improve the accuracy of detecting residual CBD stones.

There were certain disadvantages to our novel imaging technique, first that the injection of bubbles clearly has imaging consequences,which may cause false positive results. Bile should therefore be withdrawn from the ENBD tubes before initiating saline injections. US examinations are also limited to one plane, so maximum lengths are restricted accordingly. In fact, the observed lengths outside the given plane are generally longer. Dynamic observations are thus preferable. In addition, in patients with demonstrably poor US effects, as in obese subjects, ENBD-based saline-injection US is of limited utility and may increase the possibility of false negative cases.. Among all patients in this study, only three (1%) experienced discomfort upon injection, which abated once the injection was stopped. This quite possibly reflected transient scanner probe compression, which briefly elevates biliary tract pressure. All other patients were entirely asymptomatic. Furthermore, choledocholithiasis is a temporary problem. If patients get new stones during the interval period of different investigations, the imaging results will be different and cause experimental errors. In clinical situations, different examinations cannot be carried out at the same time, so this kind of error cannot be completely avoided. In this study, all of our examinations were completed within 3 days after the ERCP procedure so as to avoid the error caused by an excessively long interval time between different examinations.Finally, the use of ENBD has some drawbacks since it is uncomfortable to the patient and requires special care to avoid dislodgement,which may reduce the universality of application of ENBD-based saline-injection US.

## Conclusion

ENBD-based saline-injection US is an effective means of detecting remnant stones after ERCP. It is a simple, convenient, and easily repeatable procedure that is inexpensive and has high diagnostic sensitivity, specificity and estimated accuracy(sensitivity, 90.1%; specificity, 98.2%, estimated accuracy 96.4%). Joint use with trans-ENBD cholangiography or other existing imaging methods yields optimal results. Ultimately, its applicability for routine practice must be validated through prospective randomized controlled trials in larger patient populations.

## Supplementary Information

**Additional file1: Table S1 **Comparison of the detection rate of CBD stone with or without using lithotripsy in each method of examination

## Data Availability

The datasets generated and/or analyzed in the present study are available from the corresponding author on reasonable request.
