# Supplementary material for: Endoscopic nasobiliary drainage-based saline-injection ultrasound: an imaging technique for remnant stone detection after retrograde cholangiopancreatography
Source: BMC Gastroenterol. 2022 Jun 27;22:318. doi: 10.1186/s12876-022-02394-8 (PMC9238265; doi:10.1186/s12876-022-02394-8)
Supplement: Supplementary file 1 — Additional file1: Table S1 Comparison of the detection rate of CBD stone with or without using lithotripsy in each method of examination [file 12876_2022_2394_MOESM1_ESM.docx]

Supplementary table1: Comparison of the detection rate of CBD stone with or without using lithotripsy in each method of examination

|  | Lithotripsy | Without Lithotripsy | P value |
| --- | --- | --- | --- |
| Occlusion cholangiogram  (detection rate) | 27.8% | 10.3% | ＜0.05 |
| Routine ultrasound  (detection rate) | 11.1% | 4.3% | ＜0.05 |
| ENBD Injection ultrasound  (detection rate) | 27.8% | 16.5% | ＜0.05 |
